# Auditory Hallucinations and the Brain’s Resting-State Networks: Findings and Methodological Observations

**DOI:** 10.1093/schbul/sbw078

**Published:** 2016-06-08

**Authors:** Ben Alderson-Day, Kelly Diederen, Charles Fernyhough, Judith M. Ford, Guillermo Horga, Daniel S. Margulies, Simon McCarthy-Jones, Georg Northoff, James M. Shine, Jessica Turner, Vincent van de Ven, Remko van Lutterveld, Flavie Waters, Renaud Jardri

**Affiliations:** ^1^Psychology Department, Durham University, Durham, UK;; ^2^Department of Physiology, Development and Neuroscience, University of Cambridge, Cambridge, UK;; ^3^Department of Psychiatry, School of Medicine, University of California, San Francisco, San Francisco, CA;; ^4^New York State Psychiatric Institute, Columbia University Medical Center, New York, NY;; ^5^Max Planck Research Group for Neuroanatomy & Connectivity, Max Planck Institute for Human Cognitive and Brain Sciences, Leipzig, Germany;; ^6^Department of Psychiatry, Trinity College Dublin, Dublin, Ireland;; ^7^Mind, Brain Imaging and Neuroethics Research Unit, The Royal’s Institute of Mental Health Research, Ottawa, ON, Canada;; ^8^Department of Psychology, Stanford University, Stanford, CA;; ^9^Department of Psychology, Neuroscience Institute, Georgia State University, Atlanta, GA;; ^10^Faculty of Psychology and Neuroscience, Maastricht University, Maastricht, The Netherlands;; ^11^Center for Mindfulness, University of Massachusetts Medical School, Worcester, MA;; ^12^North Metro Health Service Mental Health, Graylands Health Campus, School of Psychiatry and Clinical Neurosciences, University of Western Australia, Crawley, WA, Australia;; ^13^Univ Lille, CNRS (UMR 9193), SCALab & CHU Lille, Psychiatry dept. (CURE), Lille, France

**Keywords:** psychosis, schizophrenia, fMRI, default mode network, perception

## Abstract

In recent years, there has been increasing interest in the potential for alterations to the brain’s resting-state networks (RSNs) to explain various kinds of psychopathology. RSNs provide an intriguing new explanatory framework for hallucinations, which can occur in different modalities and population groups, but which remain poorly understood. This collaboration from the International Consortium on Hallucination Research (ICHR) reports on the evidence linking resting-state alterations to auditory hallucinations (AH) and provides a critical appraisal of the methodological approaches used in this area. In the report, we describe findings from resting connectivity fMRI in AH (in schizophrenia and nonclinical individuals) and compare them with findings from neurophysiological research, structural MRI, and research on visual hallucinations (VH). In AH, various studies show resting connectivity differences in left-hemisphere auditory and language regions, as well as atypical interaction of the default mode network and RSNs linked to cognitive control and salience. As the latter are also evident in studies of VH, this points to a domain-general mechanism for hallucinations alongside modality-specific changes to RSNs in different sensory regions. However, we also observed high methodological heterogeneity in the current literature, affecting the ability to make clear comparisons between studies. To address this, we provide some methodological recommendations and options for future research on the resting state and hallucinations.

## Introduction

Auditory hallucinations (AH) are vivid perceptions of sound that occur without corresponding external stimuli and have a strong sense of reality. AH feature in 60%–90% of schizophrenia cases, in other psychiatric and neurological conditions, and in a minority of the general population.^1^ While many involve voices, nonverbal AH also occur (including environmental sounds, animal noises, and music).

Despite much research on the topic, many questions remain regarding the brain mechanisms of AH.^2^ One unanswered question is how they can occur spontaneously from the brain’s intrinsic activity. This has been explored by studying the brain in its so-called “resting state,” ie, the spontaneous neural activity and patterns of connectivity between brain regions that are observable when participants are asked to lie still in a scanner and not engage in any particular task.

The International Consortium on Hallucination Research (ICHR) is a global network of researchers, clinicians, and people with lived experience of hallucinations that was created to facilitate multisite collaborations.^3,4^ This ICHR report outlines our current knowledge of the resting state in relation to AH. Although elements of this topic have been reviewed elsewhere,^5,6^ this report extends prior work by incorporating evidence from a range of methods and populations, including a specific comparison of auditory and visual hallucinations. This allows us to identify the most important changes to the resting state, establishing what may be specific to AH, what may be specific to a disorder (such as schizophrenia), and what may act as a general marker for unusual perceptions across various populations. A critical review of existing methodologies and potential confounds is also presented.

Here, we first outline the general characteristics of the brain’s resting state and introduce some of the most commonly studied resting-state networks (RSNs). In the following sections, we then review functional MRI findings on RSNs relating to schizophrenia and AH and compare them with (1) evidence from other investigative methods (EEG/MEG and structural MRI) and (2) resting-state research on visual hallucinations, both in schizophrenia and in other conditions such as dementia. In the final 2 sections, we evaluate existing methodological approaches, offer a model that summarizes AH findings to date, and discuss the key issues and implications for future research.

## What Is Rest? Intrinsic Activity and Its Networks

Resting-state activity refers to the intrinsic patterns of brain activity that are observable in the absence of an external task.^7^ In fMRI, this is typically described in terms of functional connectivity: the correlations between signals in different brain regions.^8^ Spatially, the brain’s intrinsic activity can be divided into RSNs such as the default mode network (DMN), central executive network (CEN), salience network (SN), and sensorimotor networks.^9,10^ Regions involved in these RSNs (table 1) show dense functional connectivity at low frequencies (0.01–0.1 Hz) in the resting state. The DMN is often deactivated during many tasks and may be associated with self-referential or internally directed processing.^9,11^ It shows anticorrelated intrinsic activity to a collection of “task-positive” networks, including the CEN, SN, and sensorimotor networks.^12,13^ The CEN has been linked to executive functioning and cognitive control, including working memory and top-down attention.^12^ The SN has been associated with monitoring and selecting behaviorally relevant events for further processing.^14,15^ Effective goal-directed information processing may require a carefully controlled interaction between the SN and CEN, which may in turn affect processing in sensory and motor networks.^12^ Importantly though, intra- and internetwork connectivity is thought to constantly change over time.^16,17^ This generates a dynamic spatial structure to intrinsic activity, partly but not fully determined by underlying anatomy.^18,19^

**Table 1.** Common Resting-State Networks

Network	Regions	Studies in Healthy Population
Default mode network (DMN)	mPFC, precuneus, PCC, TPJ, MTL	Raichle et al^9^; Buckner et al^11^
Central executive network (CEN)	dlPFC, supragenual ACC, lateral parietal cortex	Fox et al^20^; Seeley et al^15^
Salience network (SN)	Right anterior insula, ventral striatum, dorsal ACC	Menon^10^; Goulden et al^21^
Sensorimotor networks (including language and auditory regions)	HG, left IFG, insula, bilateral STG, inferior temporal cortex, caudate, SMA	Hampson et al^22^; Beckmann et al^23^; Lee et al^24^

*Note*: ACC, anterior cingulate cortex; dlPFC, dorsolateral prefrontal cortex; HG, Heschl’s gyrus; IFG, inferior frontal gyrus; mPFC, medial prefrontal cortex; MTL, medial temporal lobe; PCC, posterior cingulate cortex; SMA, supplementary motor area; STG, superior temporal gyrus; TPJ, temporoparietal junction.

Fluctuations in electrophysiological oscillatory activity also provide a spatiotemporal structure to intrinsic activity: Functional connectivity can be assessed via the synchronization of neural oscillations between different brain regions and frequency bands such as theta (4–8 Hz), alpha (8–12 Hz), beta (12–30 Hz), and gamma (>30 Hz). However, the large majority of resting-state studies on AH have only used fMRI.^6^

## The Resting State and AH: Evidence From fMRI

AH are particularly common in schizophrenia-spectrum disorders (Sz), where they occur alongside other psychotic symptoms (such as delusions), cognitive, and functional changes. Given the primacy of AH in the disorder, RSNs of participants with schizophrenia may provide important clues to those involved in AH.

### Resting-State Findings in Schizophrenia

#### Default Mode Network.

Consistent with a cognitive profile characterized by executive dysfunction, Sz studies often show altered connectivity within the DMN and reduced anticorrelation with areas associated with the CEN, such as dorsolateral prefrontal cortex.^25^ Posterior sections of DMN in Sz also show greater connectivity to surrounding sensory areas (such as lateral occipital cortex), which may reflect problems with cognition and unusual experiences in the disorder.^26^ When DMN alterations correlate with clinical scores, they often associate with positive symptom ratings^27,28^ (for a review, see^29^).

#### Salience Network.

Striatal regions of the SN includes projections of the mesolimbic dopaminergic system (MDS), which are important in assigning novelty and significance to sensorimotor and mental events,^30^ while the anterior insula has been implicated in monitoring surprise-based prediction errors during decision-making^31^ (see Box 1). In terms of RSN dynamics, the SN has been suggested to assign salience by switching attention between the DMN and CEN.^14^ Thus, disrupted MDS activity in schizophrenia could result in atypically modulated RSNs. However, evidence of SN dysfunction specific to psychosis has been inconsistent. Two trait studies showed decreases in functional connectivity in Sz during information processing and at rest,^32,33^ and another 2 have linked connectivity alterations to general psychotic symptoms.^34,35^ Orliac et al^36^ showed that reduced connectivity in Sz between SN and DMN was linked to delusion but not hallucination severity. Negative findings of SN dysfunction also exist in Sz,^37^ although this may be due to methodological or sampling differences.

**Box 1: Predictive Coding, Auditory Hallucinations, and Rest**

A further challenge is how to integrate evidence of resting-state alterations with computational models of auditory hallucinations (AH). In parallel to the rising interest in resting-state networks, some researchers have advocated predicting processing approaches to understanding perception.^38^ Predictive coding (PC) and Bayesian models of the brain posit that perception and inference are part of a unitary process.^39,40^ Under PC, brain systems have a hierarchical organization of message passing that reduces coding of predictable information by minimizing prediction errors (PEs) in internal predictive models about the external environment. Each level of the hierarchy is comprised of functional units signaling feedback predictions and feedforward PEs, the latter being essential teaching signals that prompt updating of internal predictive models. Intuitively, PC suggests that the brain is not merely a passive feature detector, but an active creator of internal, predictive models of the environment, which determine both perception and inference about external stimuli. It follows that abnormalities in encoding of such internal predictive signals could result in abnormal percepts such as hallucinations.^41^

This framework accommodates models of dysfunctional corollary discharge in psychosis (which can be regarded as a special case of predictive coding), as well as findings of deficient mismatch negativity (MMN) in psychosis, as MMN has been considered a type of sensory PE signal.^42^ Ample evidence supports deficits in both corollary discharge mechanisms and MMN in schizophrenia^43^ with some evidence supporting their relationship to AH.^44^ Computational-model-based analyses of EEG and fMRI data have also suggested specific deficits in sensory PE signals in schizophrenia during auditory processing^45^ and in hallucinating patients with schizophrenia during speech discrimination,^46^ respectively. Deficient PEs have also been linked to increased activity in voice-selective regions of the auditory cortex,^47^ a neural phenotype previously linked to AH.^46,48,49^

According to PC and associative learning models more generally, PEs prompt learning by inducing changes in synaptic plasticity that remodel connection strengths encoding predictions.^50^ Some fMRI studies have provided evidence for connectivity changes as a function of associative learning in healthy individuals^51–53^ and for a relationship between learning and intrinsic connectivity.^54^ PC models of psychosis may therefore share a common ground with dysconnectivity views of schizophrenia, which posit that failures in synaptic plasticity (eg, NMDA-dependent plasticity and its modulation by neurotransmitters like dopamine and acetylcholine) are at the core of the disorder.^55^ Key questions to explore are how voice-selective changes to PE in auditory cortex relate to local functional connectivity within surrounding temporal cortex (which could be assessed using methods such as Regional Homogeneity) and long-range functional connectivity with default mode network, salience network (SN), and central executive network. It has also been suggested that the anterior insula, within the SN, is important for integrating interoceptive PEs that give rise to a sense of agency or presence^56^; if this were to be disrupted, it could be involved in the alterations of agency that are common in AH. Recent advances in task-based and resting-state fMRI analysis, including dynamic causal modeling,^57^ are a promising avenue to investigate the relationships between abnormal connectivity, predictive learning mechanisms, and unusual experiences such as AH.

#### Central Executive Network.

As noted above, anticorrelation between the DMN and task-oriented RSNs such as the CEN is often reduced in Sz.^29^ Consistent with executive dysfunction in Sz, resting-state connectivity increases and decreases have been reported in this network^37,58^ alongside atypical correlations with frontotemporal regions.^37^

### Resting-State Findings Specific to AH

Given DMN, SN, and CEN alterations in Sz, AH studies have focused on these RSNs, as well as on sensory networks involving auditory and language regions. Studies have either focused solely on participants with AH,^59^ compared those with and without AH,^60^ or reported correlations with hallucination severity.^61^ Few non-Sz resting-state studies of AH exist, although 2 have included people in the general community who regularly experience AH (“nonclinical voice hearers”).^62,63^

#### Default Mode Network.

Various articles have posited a specific link between DMN function and AH. For instance, Northoff and Qin^5^ proposed that resting interactions between auditory cortex and parts of the DMN may produce a state of confusion regarding external stimulation and resting-state activity. Support was provided by Jardri et al,^64^ who compared hallucinations and “real rest” periods in 20 adolescents with psychosis (participants had either AH, VH, or both). Hallucinations were associated with spontaneous engagement of sensory cortex, specific to modality, alongside disengagement and weaker integrity of the DMN (as measured by its consistency over time during scanning), suggesting that unstable DMN states may be an important precursor to AH states. Further support came from Alonso-Solis et al,^26^ who observed atypical connectivity-specific AH between hubs of the DMN and SN.

Other studies show inconsistent DMN alterations in AH. In a Sz + AH sample, Wolf et al^61^ observed no difference in DMN function or correlation with symptoms: Connectivity alterations were observed in precuneus and posterior cingulate but these were specific to an executive control network and a left frontoparietal network, respectively (see below). In contrast, van Lutterveld et al^63^ observed increased connectivity in posterior regions of the DMN in a sample of nonclinical AH participants, which may indicate differing routes to AH-proneness in clinical and nonclinical populations.

#### Central Executive Network and Salience Network.

While showing no differences in DMN function, Wolf et al^61^ observed reduced connectivity in the posterior CEN (precuneus) and increased connectivity in anterior CEN (right middle and superior frontal gyri), with increased middle frontal gyrus connectivity correlating with AH severity. Correlations with AH were also observed within a left-lateralized frontoparietal network that the authors related to speech processing and monitoring: More severe AH associated with decreased anterior cingulate cortex (ACC) connectivity and increased left superior temporal gyrus (STG) connectivity. Correlations between hallucination severity and altered resting connectivity have also been reported for the ACC,^65,66^ medial prefrontal cortex (mPFC),^59^ and anterior insula.^58^

#### Sensorimotor Networks.

Most RSN research on AH has focused on connectivity in auditory and language regions. Oertel-Knochel et al^67^ examined resting connectivity between seeds identified with an auditory language task, observing largely reduced connectivity between left auditory cortex and limbic regions in Sz + AH. Similar results were reported by Shinn et al^65^ who observed widespread reductions in connectivity for left primary auditory cortex (PAC), and by Gavrilescu et al,^68^ who observed reduced interhemispheric PAC connectivity. However, recent work by Chyzhyk et al^60^ identified right rather than left PAC as a key discriminator of AH status in Sz patients, highlighting some inconsistency in current findings.

Based on the role of Broca’s and Wernicke’s areas in speech processing, other studies have focused on connectivity between left inferior frontal gyrus (IFG) and the posterior STG, respectively. Hoffman et al^69^ observed elevated connectivity within a corticostriatal loop including STG (bilaterally), left IFG, and the putamen, in a pattern specific to Sz + AH. In contrast, Sommer et al^70^ found reduced connectivity between left IFG and left STG in AH participants, although only in comparison with healthy controls (ie, no clinical group without AH was included). In nonclinical AH, the left STG shows elevated connectivity with right STG and right IFG^62^ and acts as stronger connectivity “hub” at rest.^63^ Language lateralization may differ between clinical and nonclinical voice hearers,^71^ suggesting that extrapolating across these groups may be problematic, but taken together, these results indicate that atypical resting connectivity between left STG and other areas is common in AH.

Altogether, these studies point to a complex interaction between sensory, default mode, executive, and salience networks in AH. Inconsistent findings link overall DMN activity to AH, but there is evidence of DMN instability over time correlating with hallucination occurrence.^64^ Associations are also evident between AH and connectivity within SN and CEN, suggesting that problems with salience processing and cognitive control could contribute to a less stable balance between RSNs involved in external, sensory-guided attention.^5^ In addition, studies point to altered resting connectivity in left temporal regions implicated in auditory and language processing, although these findings require replication as some results (such as PAC connectivity) appear contradictory. In this context, drawing on evidence from other research methods, modalities, and conditions involving hallucination could help to parse out specific and general RSN properties important to AH.

## Evidence From Neurophysiology and Structural Connectivity

### From the Resting State to AH in EEG/MEG

Compared with stimulus-driven research, few EEG/MEG studies have examined the resting state in relation to either AH specifically or positive symptoms more broadly. At rest, Lee et al^72^ reported greater amplitude of beta oscillations in those with Sz + AH compared with Sz, with group differences localizing to left frontoparietal regions implicated in speech and language processing (left medial frontal gyrus and inferior parietal lobule). Andreou et al^73^ observed generally increased resting-state gamma oscillations within a left frontotemporoparietal network in Sz participants but surprisingly reduced gamma for those with higher positive symptoms (ie, those with greater levels of hallucinations and delusions had more normalized resting gamma). The actual occurrence of AH has been associated with increased gamma-theta coupling in frontotemporal areas,^74^ decreased beta power in left temporal cortex,^75^ and increased alpha connectivity between left and right auditory cortices.^76^ Analysis of rapid connectivity patterns known as “microstates” has also linked AH occurrence to shortened frontoparietal network patterns linked to error monitoring.^77^ Taken together, these findings support the primary role of left-lateralized frontotemporal cortex during AH but highlight how resting markers are likely to encompass a wider network of frontoparietal regions.

### Comparisons With Structural Connectivity

Because of the auditory-verbal nature of many AH, much research on structural connectivity has focused on integrity of the arcuate fasciculus (AF), the main white matter tract linking inferior frontal and superior temporal cortex. Consistent with RSN evidence of STG alterations, AF has generally reduced white matter integrity in Sz + AH compared with controls.^78^ There is also some evidence that this is specific to Sz + AH compared with Sz,^79^ especially for verbal AH (AVH^80^), while milder alterations to AF are evident in nonclinical voice hearers.^81^ However, as in the RSN literature, some studies have reported constrasting results, including elevated connectivity in people with Sz + AH^82^ or positive correlations between integrity and AH severity.^83^

A second tract linking STG to frontal and occipital regions (supporting a ventral rather than dorsal language pathway) is the inferior occipital-frontal fasciculus (IOFF). Two studies have reported reduced structural integrity of the left frontotemporal segment of the IOFF being specifically related to AVH.^79,84^ Consistent evidence linking AH and structural connectivity elsewhere is so far lacking. Two studies^79,85^ have reported reduced structural integrity of the corpus callosum in Sz + AH compared with Sz and healthy controls—consistent with evidence of altered interhemispheric resting connectivity in AH^68^—but an earlier study reported stronger connectivity in an Sz + AH group.^86^ There is also some evidence that integrity of the cingulum (thought to be important in DMN connectivity, eg,^87^) may be reduced in Sz + AH, although this has also been observed in Sz-only samples without any correlation to AH.^88^ No studies have specifically sought to examine structural connectivity in AH for the main RSNs discussed above. Thus, though there is emerging evidence of white matter differences beyond the AF, more evidence is needed on AH-specific white matter changes and how they may relate to resting-state pathology.

## Comparisons With VH

### Comparing the Resting State in Auditory and Visual Hallucinations in Schizophrenia

VH are roughly half as common as AH in schizophrenia^89,90^ and are more prominent in neurological conditions with known etiology (such as Lewy Body Dementia [LBD] and Charles Bonnet syndrome^91^) leading to them being less studied in Sz. Indeed, common instruments for assessing hallucinations in Sz either neglect the distinction between AH and VH^92^ or primarily focus on AH.^93^ Importantly, the majority of Sz who experience VH also experience AH, but not necessarily simultaneously, allowing for comparison between the 2 experiences.

Four studies have specifically compared Sz participants with AH and AH + VH. Because of its proposed role in salience, Rolland et al^94^ focused on connectivity of the nucleus accumbens (NAc), finding greater connectivity between NAc and bilateral insula, putamen, parahippocampal gyri, and ventral tegmental area for participants with AH + VH (compared with just AH). Meanwhile, because of its involvement in both VH^95^ and AH,^48^ Amad et al^96^ analyzed hippocampal connectivity in both groups, observing hyperconnectivity to mPFC and caudate in participants with AH + VH specifically. Those with AH + VH also had higher white matter connectivity between the hippocampus and visual cortex and greater hippocampal hypertrophy.

In contrast, Ford et al^97^ observed no differences in hippocampal connectivity between AH and AH + VH participants but reported hyperconnectivity between visual cortex and amygdala specific to those with AH + VH. In the same participants, Hare et al^98^ identified decreased amplitude of low frequency fluctuations (ALFF) in AH compared with AH + VH, in retrosplenial/inferior precuneus (BA29), left hippocampus, bilateral insula, thalamus, medial cingulate, and the medial temporal lobe. Overall, these findings show much overlap in AH and VH circuitry but also that multisensory elements involve further alterations in connectivity to limbic and striatal cortex.

### Comparisons With VH in Other Disorders

Aberrant perceptual phenomena also occur in a range of other neuropsychiatric disorders but often manifest in the visual rather than auditory domain. They are particularly common in Parkinson’s disease (PD) and LBD.^99^ Popular models implicate widespread impairments in attention and perception in the manifestation of hallucinations,^100^ which are supported by evidence from resting-state studies that show impaired connectivity within the DMN^101,102^ and between attentional networks in the brain.^103^ In addition, task-based fMRI has shown that visual misperceptions in PD are associated with increased DMN connectivity to ventral occipitotemporal regions that process object-related visual information.^104^ Interestingly, the latter results have strong convergence with the finding that the visual cortices show increased resting connectivity with the amygdala in Sz with AH + VH,^97^ suggesting similar yet divergent mechanisms underlying the manifestation of aberrant visual perception in different disorders.

Other neuropsychiatric disorders with VH demonstrate impairments in non-DMN networks. For instance, in posttraumatic stress disorder (PTSD), hallucinations and illusions (ie, misperceptions of actual stimuli) are relatively stereotyped and are closely related to the context in which the original stress occurred (eg, a war veteran with PTSD might hallucinate an enemy combatant or misperceive a telescope as a sniper rifle). Importantly, the primary pathological impairment in PTSD is related to impaired amygdala function^105^ and overactivity of regions within the ventral attention network (VAN),^106,107^ an RSN that overlaps anatomically and functionally with the SN but also typically includes regions of temporoparietal cortex.^13^ Due to this overactivity, it is assumed that any stimulus that closely matches the object related to the original stressor recruits attentional systems, leading to a rapid (and incorrect) increase in top-down influence over the ventral visual cortex, essentially “priming” the brain to incorrectly interpret the incoming stimulus. Although both LBD and PTSD are phenomenologically distinct from schizophrenia, the impairments in DMN^29^ and VAN^108^ suggest potential overlap between the different conditions and provide evidence of how hallucinations in different modalities can arise from the interaction of domain-general RSNs (managing salience and attention) with modality-specific regions.

## Current Methodology in Resting-State Studies

The study of RSNs and AH is an emerging field with a variety of often inconsistent findings. In the following section, we consider a number of potential confounds and recommendations for improving comparability and reliability of results.

### Resting-State Design and Potential Confounds

The designs of resting-state studies on AH to date have been far from uniform (see table 2), which hampers comparison of study findings. In table 3, we offer some suggestions to improve uniformity across experiments, some of which are specific to AH research and some that are more general. Most important in AH studies are participant instructions and debriefing: Instructing participants to keep their eyes closed is associated with higher functional connectivity within auditory networks^109^ (which may not be desirable) and arguably increases the likelihood of participants falling asleep. We therefore suggest that future studies use an eyes-open design. Beyond this, instructing participants to do anything other than relax can lead to signal changes (eg,^110^) and can create demand characteristics: When participants know in advance that they will be asked about AH, this can induce attention effects which confound hallucination-related brain activity.^111^ A thorough debrief about unusual experiences after scanning is preferred to online monitoring.

**Table 2.** Study Characteristics of Resting-State fMRI Studies Into AH

Study	EO/EC	Scan Length (min)	RS Instructions	Presence of AH Asked?
Alonso-Solis et al^26^	EC	6	Pts instructed to close eyes and remain awake	Not reported
Chyzhyk et al^60^	EO	10	Stay awake, keep eyes open, and think of nothing in particular	Yes for some participants
Clos et al^59^	EC	6	Lie in the scanner as still as possible with their eyes closed yet stay awake	Yes
Diederen et al^62^	EC	6	Pts kept eyes closed but stayed awake	Yes
Gavrilescu et al^68^	EC	5	Relax with eyes closed	Yes
Jardri et al^64^	EC	15	Pts kept still in a state of wakeful rest with eyes closed	Yes
Manoliu et al^58^	EC	10	Eyes closed and not to fall asleep	Pts asked about any “feelings of odd situations” during scan.
Oertel-Knochel et al^67^	EO	6.7	Lie still, do not engage in any speech, think nothing specially and look at white fixation cross	Yes
Rotarska-Jagiela et al^28^	EO	6.7	Lie still with eyes open fixating on a white cross presented in the center of visual field.	Not reported
Shinn et al^65^	EO	10	Stay awake, keep eyes open, and think of nothing in particular	Yes for some participants
Sommer et al^70^	EC	6	Pts instructed to lie in scanner as still as possible with eyes closed yet stay awake	Yes
Sorg et al^112^	EC	10	Keep eyes closed and not to fall asleep.	Pts asked about any “feelings of odd situations” during scan.
Van Lutterveld et al^63^	EC	6	Pts kept eyes closed but stayed awake	Yes
Vercammen et al^66^	EC	7.8	Close eyes and try to “clear your mind” but not fall asleep.	No
Wolf et al^61^	EC	6	Relax without falling asleep, keep eyes closed, not think about anything in particular, and move as little as possible	Yes

*Note*: AH, auditory hallucinations; EC, eyes closed; EO, eyes open; Pts, participants. Hoffman et al^69^ extracted intermittent resting data from a symptom capture paradigm involving button pressing, and so is not included here.

**Table 3.** Recommended Methods for Resting Studies of AH

Basic demographics and pre-interview	Age, gender, sleep patterns, nicotine use, medication, hallucination phenomenology
Participant instructions	Eyes open, relax, keep still. Emphasize not falling asleep
Stimuli	Fixation cross on gray, nonbright background (if eyes open)
Scan length	At least 6min; over 10min preferred
Concurrent measures	Head movements, cardiorespiratory signal, sleep monitoring (camera or concurrent EEG)
Debrief	Presence of hallucinations during scanning, emotional state (eg, anxiety), other unusual experiences
Analysis	Seed-based (for hypothesis-testing); common seeds include IFG, STG, TPJ, ACC, hippocampus, insula. ICA-based (preferred); allows analysis of network interactions. Graph-theoretical measures (eg, path- length, betweenness centrality)
Common network frameworks	Triple network (DMN/CEN/SN^10^) or Yeo et al^113^ 7 or 17 network solution

*Note*: Abbreviations are explained in the first footnote to table 1. AH, AH, auditory hallucinations; CEN, central executive network; DMN, default mode network; ICA, independent component analysis; SN, salience network.

In general, longer scan times are preferable (increasing scan time to 13min greatly improves reliability^114^), and it is important to control for potential effects of head movement, cardiac rate, and respiration.^115–117^ Data scrubbing of bad volumes^118^ or matching groups for head motion are 2 ways of counteracting potential movement effects. Finally, during data analysis, use of global signal regression to account for nonneuronal artifacts should be avoided, as this can introduce spurious negative correlations.^119^

### Resting-State Analysis

There are 2 common approaches to analyzing resting-state fMRI connectivity data: strongly and weakly model-driven. In seed-regression analyses, correlations are calculated between an a priori selected region and the rest of the brain. This is a strongly model-driven approach, as the selected seed region represents a spatial hypothesis about the brain state of interest, and is the most common approach used in resting-state AH studies. In contrast, 2 other methods have been used to characterize functional brain dynamics in a multivariate, weakly model-driven approach: independent component analysis (ICA) and graph theory. ICA is a multivariate, data-driven analysis tool of the Blind Source Separation family,^120^ which has been shown to be particularly well-suited for analyzing distributed networks of intrinsic brain activity.^121^ Unlike seed-based analysis, ICA does not require a priori definition of a seed region or ideal model of activity and provides multiple networks in a single analysis. Moreover, it has been argued that ICA has better test-retest reliability than seed-based methods^122^ and may be better at estimating functional statistical maps in the case of unpredictable events such as hallucinations without the need for signaling events’ occurrences online.^64,123^ Crucially, when participants hallucinate during a scan, ICA can be used to capture both DMN and hallucinatory fMRI activity: Jardri et al^64^ describe a validated procedure for doing so that uses retrospective AH reports from participants. This property of ICA makes possible the exploration of the interplay between DMN and sensory cortices over time during “rest” and “hallucination” periods (defined at the individual IC level), without disrupting intrinsic RSN behavior. Finally, graph theory mathematically describes the architecture of interregional connections (ie, edges) of multiple brain areas (ie, nodes) in relation to efficiency of information processing,^124^ which can be used to characterize local or global changes in network architecture. Graph theory can be considered complementary to the other functional connectivity tools, as it is often applied to output of the seed-based or ICA approaches. Its value is in allowing investigators to go beyond simple correlations between regions to examine the relative importance of paths and hubs within a network.

## AH and the Resting State: Key Issues and Implications

### RSNs and AH: A Synthesis of Findings

The investigation of RSNs opens up a range of opportunities for understanding how and why hallucinations occur. The majority of AH research has so far focused on intrinsic connectivity of regions associated with speech, language, and auditory processing, both in fMRI and EEG/MEG: Most consistent are findings of altered connectivity in left posterior temporal regions implicated in speech perception (which are supported by structural evidence), but there are also multiple studies implicating inferior frontal, parietal, limbic, and striatal regions.^6^ Such findings broadly support theories that posit disruptions to internal speech and language processes,^125^ although not consistently (cf.^69,70^). This is complemented by a growing body of evidence linking the DMN, CEN, and SN to both AH occurrence and predisposition, lending support to models of hallucinations that go beyond speech processes and emphasize other factors, such as cognitive control and attention.^126^ As outlined in prior ICHR collaborations,^127^ it is generally accepted that AH are likely to involve multiple cognitive mechanisms in their development, suggesting that a focus on multiple brain networks is required.

**Fig. 1.**
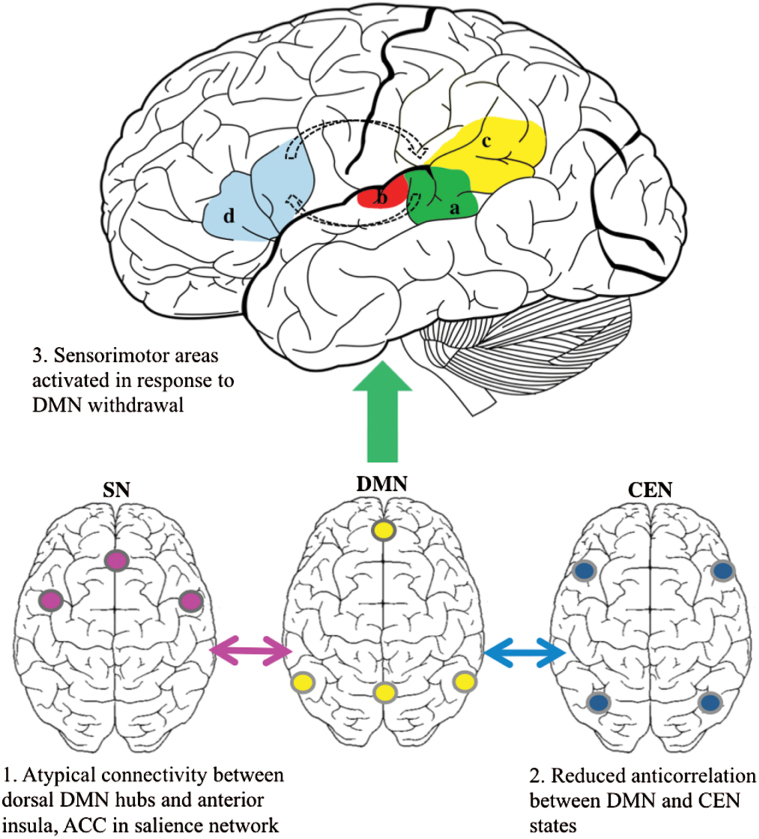
Initial AH studies focused on resting connectivity in auditory and language regions (upper figure), primarily identifying atypical connectivity of left posterior STG (a), PAC (b), and the TPJ area (c). Findings of atypical resting connectivity between left IFG (d) and STG are inconsistent although both areas are often implicated during AH. More recent findings implicate atypical interaction of the DMN, SN, and CEN in those prone to AH (lower figures). The combination of atypical DMN interaction with SN (1) and CEN (2) and altered resting connectivity in sensory areas could prompt the collapse of internally focused states into activation of auditory cortex (3), which is then reverberated along a frontotemporal loop. The IFG, STG, and surrounding areas are often implicated in symptom-capture studies.^48^ *Note*: ACC, anterior cingulate cortex; AH, auditory hallucination; CEN, central executive network; DMN, default mode network; IFG, inferior frontal gyrus; PAC, primary auditory cortex; SN, salience network; STG, superior temporal gyrus; TPJ, temporoparietal junction.

Few studies have demonstrated specific trait associations between DMN properties and AH (in comparison with evidence of general links to positive symptoms, eg,^25^). However, the interaction of the DMN with other RSNs^26,58^ and dynamic stability of the DMN^64^ are both implicated in hallucinatory states and traits. It is possible that atypical interactions of the DMN, SN, and CEN, when *allied* with altered resting connectivity in language and sensory networks, give rise to the collapse of internal states into sensory processing as described by Jardri et al^64^ (see figure 1). This also partly supports Northoff and Qin’s^5^ proposal that AH arise from elevated resting-state activity in auditory areas and irregular modulation by anterior hubs of the DMN: The evidence here does not suggest a direct link between them but implies atypical DMN-SN-CEN interaction *alongside* connectivity alterations to sensory cortices. However, these findings are clearly preliminary and require further testing, especially in the face of inconsistent results and methodological heterogeneity. It will be important to examine how hubs of the domain-general RSNs interact with auditory areas, and the future use of alternative seeds/ROIs or ICA will shed further light on how these other networks contribute to AH.

Comparisons with mechanisms in VH provide an important source of information. Evidence of DMN instability in both AH and VH in psychosis and disruptions to DMN, executive, and salience networks in VH in other disorders imply a similar framework for hallucination susceptibility across different modalities. However, contrasts between AH and AH + VH in schizophrenia also suggest that disruptions to limbic and striatal connectivity may distinguish trait susceptibility for these experiences,^96,97^ suggesting that more complex hallucinatory phenomena may require a higher base level of resting connectivity between sensory and limbic regions. Comparison with VH raises the question of whether other RSNs need to be examined in more detail in AH: eg, while a number of AH and Sz studies have focused on the SN, the VAN is relatively unstudied. The SN and VAN overlap in many ways—and the exact relationship between them is still unclear—but definitions of VAN often emphasize right frontoparietal structures (such as IFG and TPJ) rather than just insular and cingulate cortex (eg,^13,128^). Given that functional and structural characteristics of these regions have been related to AH occurrence and phenomenology,^129,130^ the VAN may prove a fruitful avenue for further investigation.

Indeed, the current focus on DMN, CEN, and SN in AH research arguably reflects only one approach to RSNs—the “triple network” model.^10^ Other network approaches, such as that used by Yeo et al,^113^ use many more functional networks in parallel. Future AH studies may choose to adopt such broader network solutions, especially to allow comparison with research on healthy cognitive function. Understanding of the DMN and other RSNs is also constantly advancing: The early discovery that the DMN is negatively correlated with another set of regions—namely those of the CEN^20^—was called into question by work demonstrating variability in this negative relationship over time.^131^ Several subsequent studies have further revealed the complex spatiotemporal patterns of activity that underlie RSNs (eg,^132–134^ for a review see^16^), while task studies show that regions distributed across the whole brain and multiple networks are often involved in “single” processes.^135^ In this respect, it is important to recognize that terms such as DMN or CEN are only placeholders for complex and likely cross-network processes, and researchers should be generally wary of treating them as modular, insulated entities.

### Increasing Study Reliability and Aggregating Data

In moving forward with resting-state research on AH, improving the comparability and reliability of results is a clear priority. One way of doing this is following a shared “standard” protocol (such as in table 3); another, more powerful approach is to aggregate data across studies, facilitating the acquisition of a more heterogeneous and representative subject sample.^136^ fMRI data are typically pooled using meta-analyses of significant activations (eg,^137^), but such analyses cannot aggregate power across studies as they rarely report effect sizes. A more direct method for aggregating data is by combining (raw) data across centers, also referred to as mega-analysis.^138^ Although the instruction and fMRI protocol may differ across studies, this approach allows for the use of the same preprocessing and analysis on all data. Another option consists of multicenter studies in which the scan protocol, participant instructions, scanner, software, hardware, etc. are standardized.^139^ Variability between centers can be monitored through the use of acquisition of phantom data and travelling heads, ie, control subjects that are scanned at each site to estimate cross-site variability. Although recent studies indicate that scanner-related variance is low compared with between-subject variability and measurement error,^140,141^ it can be modeled by entering study site as a random effects factor capturing variability between study sites.^142^ In this vein, the authors of this article are currently planning a mega-analysis of existing resting-state data from research teams within the ICHR: To date, 7 participating centers have agreed to aggregate their data (which includes over 150 participants with AH).

### Clinical Implications and Directions for Further Research

As a young field, resting-state research on AH is still at the stage of basic rather than translational science. However, the opportunity to study state and trait markers of AH using resting methods has great clinical potential: In a recent example, Mondino et al^143^ demonstrated changes in resting temporoparietal connectivity associated with symptom reduction in patients who had completed 10 weeks of transcranial direct current stimulation for persistent AH. A further way of making resting-state studies more clinically relevant would be to include more detailed examination of hallucinatory phenomenology and contextual factors, both in general and following scanning. While many studies have reported AH symptom correlations, this is usually a total severity score, rather than specific to different kinds of phenomenological features, and wider environmental and social variables are almost never included. Other key remaining questions, with basic and applied implications, are how resting-state fMRI findings relate to functional connectivity measured with neurophysiological methods (eg,^144^), evidence of changes to anatomical connectivity,^78^ and computational models of hallucinations, such as predictive processing approaches (see Box 1). The use of multimethod studies will go some way to address these issues in future research.

## Conclusion

The spatiotemporal dynamics of the brain’s resting state have great potential to offer new insights in the study of hallucinations. Studies of AH highlight altered resting connectivity of speech and language regions, such as the left STG, although there is growing evidence of domain-general resting networks (and thus the brain’s spontaneous activity as a whole) being implicated in Sz + AH and perhaps other kinds of hallucinatory states. Studies of VH suggest that additional networks may be involved, including limbic and striatal regions, although more studies contrasting RSN in VH and AH are needed across different population groups. Alongside this, the study of RSNs is subject to a range of potential confounds and methodological differences, prompting a need to combine efforts and methods across laboratories. A more connected approach will improve comparability and reliability of studies and enhance understanding of how, in the case of AH, a signal may emerge from noise.

## Funding

B.A.-D. and C.F. are supported by Wellcome Trust (WT098455); C.F. and D.S.M. by Wellcome Trust (WT103817).
